# Supplementary figures and images for: The mitochondrial genome sequences of eleven leafhopper species of Batracomorphus (Hemiptera: Cicadellidae: Iassinae) reveal new gene rearrangements and phylogenetic implications
Source: PeerJ. 2024 Oct 22;12:e18352. doi: 10.7717/peerj.18352 (PMC11505954; doi:10.7717/peerj.18352)

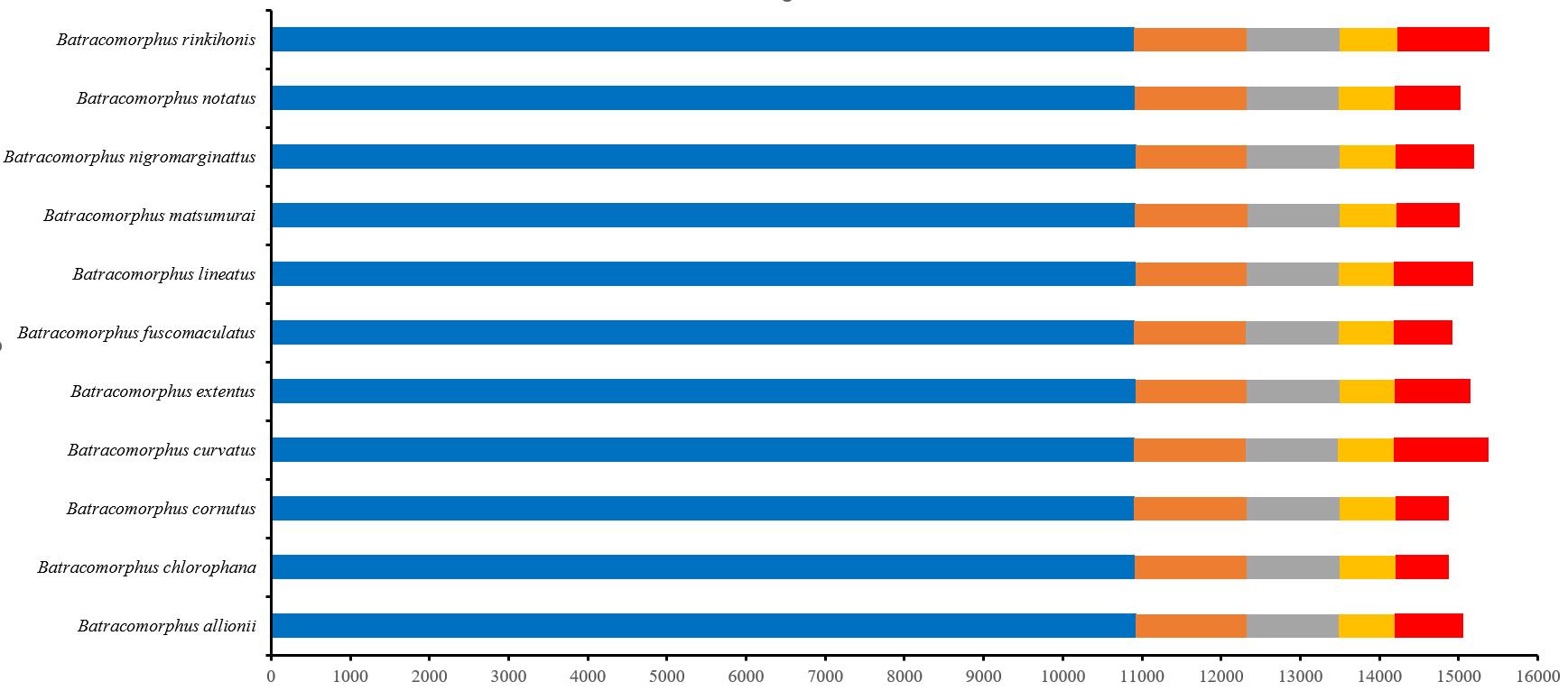

Supplement: Figure S1 [file peerj-12-18352-s001.jpg]

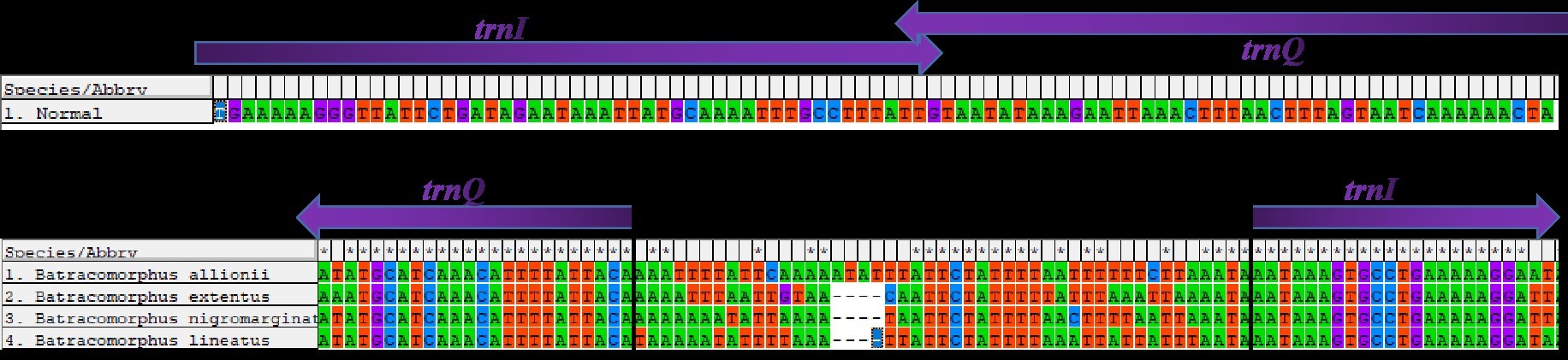

Supplement: Figure S2 [file peerj-12-18352-s002.jpg]
