# Supplementary material for: The mitochondrial genome sequences of eleven leafhopper species of Batracomorphus (Hemiptera: Cicadellidae: Iassinae) reveal new gene rearrangements and phylogenetic implications
Source: PeerJ. 2024 Oct 22;12:e18352. doi: 10.7717/peerj.18352 (PMC11505954; doi:10.7717/peerj.18352)
Supplement: Table S1 [file peerj-12-18352-s008.docx]

Table S1. Sources and information used for the polygenomic analysis.

| **Subfamily** | **Species** | **Size (bp)** | **Accession number** | **Source** |
| --- | --- | --- | --- | --- |
| Deltocephalinae | *Drabescoides nuchalis* | 15,309 | NC_028154 | Wu et al. 2016 |
|  | *Japananus hyalinus* | 15,364 | NC_036298 | Du et al. 2017 |
|  | *Macrosteles quadrilineatus* | 16,626 | NC_034781 | Mao et al. 2017 |
|  | *Maiestas dorsalis* | 15,352 | NC_036296 | Du et al. 2017 |
|  | *Phlogotettix* sp. | 15,136 | KY039135 | Song et al. 2017 |
|  | *Scaphoideus maai* | 15,188 | KY817243 | Du et al. 2017 |
|  | *Scaphoideus varius* | 15,207 | KY817245 | Du et al. 2017 |
|  | *Scaphoideus nigrivalveus* | 15,235 | KY817244 | Du et al. 2017 |
|  | *Alobaldia tobae* | 16,026 | KY039116 | Unpublished |
|  | *Paramacrosteles nigromaculatus* | 15,011 | NC_045270 | Yang et al. 2019 |
|  | *Roxasellana* *stellata* | 15,361 | NC_050257 | Xu et al. 2020 |
|  | *Hishimonoides recurvatis* | 14,814 | KY364883 | Unpublished |
|  | *Norvellina* sp. | 15,594 | KY039131 | Unpublished |
|  | *Tambocerus* sp. | 15,955 | KT827824 | Yu et al. 2017 |
|  | *Paradorydium reflexanum* | 15,661 | MG813487 | Wang et al. 2018 |
|  | *Cicadula* sp. | 14,929 | KX437724 | Song et al. 2018 |
|  | *Exitianus indicus* | 16,089 | KY039128 | Song et al. 2017 |
|  | *Orosius orientalis* | 15,513 | KY039146 | Song et al. 2017 |
|  | *Paralaevicephalus gracilipenis* | 16,114 | MK450366 | Wang et al. 2019 |
|  | *Yanocephalus yanonis* | 15,623 | NC_036131 | Song et al. 2017 |
|  | *Macrosteles quadrimaculatus* | 15,734 | NC_039560 | Du et al. 2018 |
|  | *Abrus expansivus* | 15,904 | NC_045238 | Wang et al. 2019 |
|  | *Drabescus ineffectus* | 15,744 | NC_050258 | Xu et al. 2020 |
|  | *Reticuluma hamata* | 15,190 | NC_051985 | Xu er al. 2021 |
|  | *Phlogotettix monozoneus* | 15,199 | NC_056240 | Unpublished |
|  | *Fieberiella septentrionalis* | 16,175 | NC_057252 | Unpublished |
| Mileewinae | *Mileewa albovittata* | 15,079 | MK138358 | He et al. 2019 |
|  | *Mileewa alara* | 16,020 | MW533151 | He et al. 2019 |
|  | *Mileewa margheritae* | 15,375 | MT483998 | He et al. 2019 |
|  | *Mileewa ponta* | 15,999 | MT497465 | Unpublished |
| Cicadellinae | *Bothrogonia tongmaiana* | 15,539 | NC_049895 | Unpublished |
|  | *Bothrogonia ferruginea* | 15,262 | KU167550 | Unpublished |
|  | *Bothrogonia qiongana* | 15,788 | NC_049894 | Xu et al. 2020 |
|  | *Bothrogonia shuana* | 15,229 | NC_060293 | Xu et al. 2020 |
|  | *Cicadella viridis* | 15,891 | MK335936 | Zhong et al. 2019 |
|  | *Kolla paulula* | 14,995 | MW542170 | Unpublished |
|  | *Anagonalia melichari* | 15,398 | MT642611 | Unpublished |
|  | *Atkinsoniella aurantiaca* | 15,394 | NC_062843 | Jiang et al. 2022 |
|  | *Atkinsoniella grahami* | 15,621 | MW533712 | Jiang et al. 2021 |
|  | *Gunungidia aurantiifasciata* | 15,472 | NC_056102 | Zhang et al. 2020 |
|  | *Cofana yasumatsui* | 15,019 | NC_049087 | Zhong et al. 2020 |
| Coelidiinae | *Olidiana ritcheriina* | 15,166 | NC_045207 | Wang et al. 2019 |
|  | *Olidiana lonisticka* | 15,993 | NC_057963 | Wang et al. 2021 |
|  | *Olidiana obliquea* | 15,312 | NC_057964 | Wang et al. 2021 |
|  | *Olidiana ritcheri* | 15,372 | NC_057965 | Wang et al. 2021 |
|  | *Olidiana tongmaiensis* | 15,363 | NC_057966 | Wang et al. 2021 |
|  | *Taharana fasciana* | 15,161 | KY886913 | Wang et al. 2019 |
| Iassinae | *Trocnadella arisana* | 15,131 | NC_036480 | Wang et al. 2020a |
|  | *Gessius rufidorsus* | 15,423 | MN577633 | Wang et al. 2020a |
|  | *Batracomorphus lateprocessus* | 15,356 | NC_045858 | Wang et al. 2020a |
|  | *Batracomorphus fuscomaculatus* | 14,915 | 0Q873422 | This study |
|  | *Batracomorphus notatus* | 15,016 | 0Q873426 | This study |
|  | *Batracomorphus chlorophana* | 14,870 | 0Q873418 | This study |
|  | *Batracomorphus cornutus* | 15,022 | 0Q873419 | This study |
|  | *Batracomorphus rinkihonis* | 15,385 | 0Q873427 | This study |
|  | *Batracomorphus allionii* | 15,026 | 0Q873417 | This study |
|  | *Batracomorphus nigromarginattus* | 15,185 | 0Q873425 | This study |
|  | *Batracomorphus lineatus* | 15,174 | 0Q873423 | This study |
|  | *Batracomorphus extentus* | 15,137 | 0Q873421 | This study |
|  | *Batracomorphus matsumurai* | 15,356 | 0Q873424 | This study |
|  | *Batracomorphus curvatus* | 15,009 | 0Q873420 | This study |
|  | *Krisna concava* | 14,304 | NC_046067 | Wang et al. 2020a |
|  | *Krisna rufimarginata* | 14,724 | NC_046068 | Wang et al. 2020a |
| Eurymelinae | *Idiocerus laurifoliae* | 16,811 | MH433622 | Wang et al. 2018 |
|  | *Populicerus populi* | 16,494 | MH492318 | Wang et al. 2018 |
|  | *Populicerus confusus* | 16,395 | NC_050982 | Shan et al. 2020 |
|  | *Idiocerus herrichii* | 15,489 | MN935487 | Shan et al. 2020 |
|  | *Rhytidodus viridiflavus* | 16,842 | MN935488 | Di et al. 2020 |
|  | *Metidiocerus impressifrons* | 16,426 | MW963341 | Unpublished |
|  | *Koreocerus koreanus* | 16,428 | MZ169558 | Unpublished |
|  | *Idiocerus salicis* | 16,436 | NC_046048 | Wang et al. 2018 |
|  | *Idioscopus clypealis* | 15,393 | NC_039642 | Wang et al. 2018 |
|  | *Oncopsis nigrofasciata* | 15,927 | MG813492 | Wang et al. 2020 |
|  | *Macropsis notata* | 16,323 | NC_042723 | Wang et al. 2020 |
| Megophthalminae | *Durgades nigropicta* | 15,974 | NC_035684 | Wang et al. 2017 |
|  | *Japanagallia spinosa* | 15,655 | NC_035685 | Wang et al. 2017 |
| Typhlocybinae | *Parathailocyba orla* | 15,382 | MN894531 | Jiang et al. 2020 |
|  | *Mitjaevia protuberanta* | 15,472 | NC_047465 | Yuan et al. 2020 |
|  | *Empoascanara dwalata* | 15,271 | MT350235 | Unpublished |
|  | *Eupteryx adspersa* | 15,178 | MZ014454 | Lin et al. 2021 |
|  | *Kaukania anser* | 15,345 | MZ014456 | Lin et al. 2021 |
|  | *Aguriahana digitata* | 15,854 | MZ457330 | Unpublished |
|  | *Paraahimia luodianensis* | 16,497 | NC_047464 | Song et al. 2020 |
|  | *Parazyginella tiani* | 17,562 | NC_053918 | Zhou et al. 2020 |
|  | *Dikraneura zlata* | 15,330 | MZ014450 | Lin et al. 2021 |
|  | *Limassolla lingchuanensis* | 15,716 | NC_046037 | Yuan et al. 2020 |
|  | *Zyginella minuta* | 15,544 | NC_052876 | Han et al. 2020 |
|  | *Bolanusoides shaanxiensis* | 15,274 | MN661136 | Unpublished |
| Ledrinae | *Ledra trigona* | 16,094 | MG813491 | Wang et al. 2019 |
|  | *Petalocephala eurglobata* | 14,834 | MW018817 | Unpublished |
|  | *Petalocephala_chlorophana* | 14,927 | NC_051527 | Huang et al. 2020 |
|  | *Petalocephala gongshanensis* | 14,969 | MW018818 | Unpublished |
|  | *Tituria pyramidata* | 15,331 | NC_046701 | Unpublished |
|  | *Tituria sagittata* | 14,918 | NC_051528 | Unpublished |
| Hylicinae | *Hylica paradoxa* | 14,762 | NC_056920 | Tang et al. 2020 |
|  | *Balala fujiana* | 16,221 | NC_056921 | Tang et al. 2020 |
|  | *Kalasha nativa* | 15,716 | NC_056922 | Tang et al. 2020 |
|  | *Nacolus tuberculatus* | 15,737 | NC_056923 | Tang et al. 2020 |
| Evacanthinae | *Chudania hellerina* | 15,044 | MN227164 | Du et al. 2021 |
|  | *Concaveplana rufolineata* | 15,240 | MN227168 | Du et al. 2021 |
|  | *Evacanthus danmainus* | 15,343 | MN227166 | Du et al. 2021 |
| Treehoppers | *Maurya qinlingensis* | 16,011 | NC_044706 | Hu et al. 2019 |
|  | *Tricentrus brunneus* | 16,467 | NC_044708 | Hu et al. 2019 |
|  | *Leptocentrus albolineatus* | 15,508 | NC_044707 | Hu et al. 2019 |
|  | *Hypsauchenia hardwickii* | 15,618 | NC_044705 | Hu et al. 2019 |
|  | *Entylia carinata* | 15,662 | NC_033539 | Mao et al. 2016 |
| Cicadoidea | *Tettigades auropilosa* | 14,944 | KM000129 | Unpublished |
| Cicadoidea | *Diceroprocta semicincta* | 14,920 | KM000131 | Unpublished |
|  | *Magicicada tredecim* | 14,435 | NC_041652 | Du et al. 2019 |
|  | *Aeneolamia contigua* | 15,613 | NC_025495 | Liu et al. 2014 |
|  | *Philaenus spumarius* | 16,324 | NC_005944 | Stewart et al. 2018 |
| Cercopoidea | *Cosmoscarta bispecularis* | 15,426 | KP064511 | Yang et al. 2014 |
