# Supplementary material for: The mitochondrial genome sequences of eleven leafhopper species of Batracomorphus (Hemiptera: Cicadellidae: Iassinae) reveal new gene rearrangements and phylogenetic implications
Source: PeerJ. 2024 Oct 22;12:e18352. doi: 10.7717/peerj.18352 (PMC11505954; doi:10.7717/peerj.18352)
Supplement: Table S2 [file peerj-12-18352-s009.docx]

**Table S2. Base content and skew of various types of mitochondrial genes in the *Batracomorphus***

| **Species** |  | **Length (bp)** | **A%** | **C%** | **G%** | **T%** | **AT%** | **AT Skew** | **GC Skew** |
| --- | --- | --- | --- | --- | --- | --- | --- | --- | --- |
| *Batracomorphus* *allionii* | PCG | 10,919 | 36.00 | 10.90 | 9.60 | 43.50 | 79.40 | -0.09 | 0.06 |
|  | tRNA | 1,403 | 39.90 | 9.00 | 12.30 | 38.80 | 78.70 | 0.01 | 0.15 |
|  | rRNA | 1,874 | 33.90 | 6.40 | 10.70 | 49.00 | 82.90 | -0.18 | 0.25 |
|  | CK | 801 | 47.2 | 8.5 | 5.9 | 38.5 | 85.70 | 0.10 | -0.18 |
|  | Whole | 15025 | 46.8 | 11.6 | 8.2 | 33.4 | 80.20 | 0.17 | -0.17 |
| *Batracomorphus* *chlorophana* | PCG | 10,904 | 34.80 | 10.90 | 10.10 | 44.20 | 79.00 | -0.12 | 0.04 |
|  | tRNA | 1,420 | 40.80 | 8.20 | 12.00 | 39.10 | 79.90 | 0.02 | 0.19 |
|  | rRNA | 1,886 | 31.80 | 6.70 | 10.80 | 50.70 | 82.50 | -0.23 | 0.23 |
|  | CK | 698 | 48.4 | 7.3 | 5.9 | 38.4 | 86.80 | 0.12 | -0.11 |
|  | Whole | 14870 | 47.4 | 11.9 | 8.2 | 32.6 | 80.00 | 0.19 | -0.18 |
| *Batracomorphus cornutus* | PCG | 10,898 | 35.20 | 10.40 | 9.70 | 44.70 | 79.90 | -0.12 | 0.03 |
|  | tRNA | 1,419 | 41.30 | 8.50 | 11.80 | 38.50 | 79.80 | 0.04 | 0.16 |
|  | rRNA | 1,888 | 32.90 | 6.50 | 10.80 | 49.80 | 82.70 | -0.20 | 0.25 |
|  | CK | 832 | 48 | 5.8 | 5.6 | 40.6 | 88.60 | 0.08 | -0.02 |
|  | Whole | 15021 | 47.1 | 11.2 | 8.1 | 33.6 | 80.70 | 0.17 | -0.16 |
| *Batracomorphus curvatus* | PCG | 10,900 | 34.90 | 10.40 | 9.80 | 44.90 | 79.80 | -0.13 | 0.03 |
|  | tRNA | 1,409 | 40.90 | 7.90 | 11.40 | 39.70 | 80.60 | 0.01 | 0.18 |
|  | rRNA | 1,880 | 33.88 | 6.60 | 10.30 | 49.30 | 83.28 | -0.19 | 0.22 |
|  | CK | 1212 | 45.6 | 10.4 | 6.6 | 37.4 | 83.00 | 0.10 | -0.22 |
|  | Whole | 15347 | 46.3 | 11.3 | 8.2 | 34.2 | 80.50 | 0.15 | -0.16 |
| *Batracomorphus* *extentus* | PCG | 10,916 | 35.40 | 11.20 | 10.30 | 43.30 | 78.60 | 0.10 | -0.04 |
|  | tRNA | 1,409 | 40.50 | 8.60 | 12.20 | 38.70 | 79.20 | 0.02 | 0.17 |
|  | rRNA | 1,873 | 33.60 | 6.40 | 10.70 | 49.40 | 83.00 | -0.19 | 0.25 |
|  | CK | 886 | 47.2 | 7.4 | 6.0 | 39.4 | 86.60 | 0.09 | -0.10 |
|  | Whole | 15138 | 47.5 | 12.1 | 8.2 | 32.2 | 79.70 | 0.19 | -0.19 |
| *Batracomorphus fuscomaculatus* | PCG | 10,898 | 35.40 | 10.50 | 9.60 | 44.50 | 79.90 | -0.11 | 0.04 |
|  | tRNA | 1,415 | 41.60 | 8.10 | 11.70 | 38.60 | 80.20 | 0.04 | 0.18 |
|  | rRNA | 1,872 | 32.30 | 6.30 | 10.70 | 50.70 | 83.00 | -0.22 | 0.26 |
|  | CK | 737 | 47.5 | 7.1 | 6.4 | 39.1 | 86.60 | 0.10 | -0.05 |
|  | Whole | 14913 | 47.5 | 11.4 | 8.0 | 33.2 | 80.70 | 0.18 | -0.18 |
| *Batracomorphus lineatus* | PCG | 10,918 | 35.20 | 11.60 | 10.40 | 42.70 | 77.90 | -0.10 | 0.05 |
|  | tRNA | 1,400 | 40.10 | 8.40 | 12.10 | 39.40 | 79.50 | 0.01 | 0.18 |
|  | rRNA | 1,872 | 33.40 | 6.60 | 10.50 | 49.60 | 82.90 | -0.20 | 0.23 |
|  | CK | 946 | 47.4 | 8.1 | 5.0 | 39.5 | 86.90 | 0.09 | -0.24 |
|  | Whole | 15174 | 46.6 | 112.1 | 8.6 | 32.7 | 79.30 | 0.18 | -0.86 |
| *Batracomorphus matsumurai* | PCG | 10,908 | 34.70 | 10.60 | 9.90 | 44.80 | 79.50 | -0.13 | 0.03 |
|  | tRNA | 1,422 | 40.60 | 7.90 | 11.40 | 40.10 | 80.70 | 0.01 | 0.18 |
|  | rRNA | 1,886 | 33.60 | 6.50 | 10.50 | 49.40 | 83.00 | -0.19 | 0.24 |
|  | CK | 822 | 46.5 | 9.1 | 6.3 | 38.1 | 84.60 | 0.10 | -0.18 |
|  | Whole | 15008 | 46.4 | 11.5 | 8.2 | 33.9 | 80.30 | 0.16 | -0.17 |
| *Batracomorphus* *nigromarginattus* | PCG | 10,919 | 36.40 | 10.40 | 9.50 | 43.70 | 80.10 | -0.09 | 0.05 |
|  | tRNA | 1,404 | 40.50 | 8.88 | 11.50 | 39.20 | 79.70 | 0.02 | 0.13 |
|  | rRNA | 1,885 | 33.30 | 6.40 | 10.30 | 50.10 | 83.40 | -0.20 | 0.23 |
|  | CK | 931 | 47.5 | 8.3 | 4.6 | 39.6 | 87.10 | 0.09 | -0.29 |
|  | Whole | 15183 | 48 | 11.2 | 7.8 | 33.0 | 81.00 | 0.19 | -0.18 |
| *Batracomorphus notatus* | PCG | 10,904 | 35.10 | 10.40 | 10.00 | 44.50 | 79.60 | -0.12 | 0.02 |
|  | tRNA | 1,415 | 41.10 | 8.00 | 12.30 | 38.70 | 79.80 | 0.03 | 0.21 |
|  | rRNA | 1,872 | 31.60 | 6.70 | 10.70 | 51.00 | 82.60 | -0.23 | 0.23 |
|  | CK | 858 | 47.7 | 6.1 | 8.3 | 38 | 85.70 | 0.11 | 0.15 |
|  | Whole | 15015 | 47.5 | 11.4 | 8.3 | 32.8 | 80.30 | 0.18 | -0.16 |
| *Batracomorphus rinkihonis* | PCG | 10,901 | 35.40 | 10.20 | 9.80 | 44.60 | 80.00 | 0.12 | -0.02 |
|  | tRNA | 1,420 | 41.70 | 8.00 | 11.50 | 38.80 | 80.50 | 0.04 | 0.18 |
|  | rRNA | 1,910 | 33.20 | 6.40 | 10.30 | 50.20 | 83.40 | -0.21 | 0.23 |
|  | CK | 1179 | 44.9 | 7.9 | 8.2 | 39 | 83.90 | 0.07 | 0.02 |
|  | Whole | 15384 | 47.3 | 11.2 | 8.0 | 33.5 | 80.80 | 0.17 | -0.17 |
