# Supplementary material for: The mitochondrial genome sequences of eleven leafhopper species of Batracomorphus (Hemiptera: Cicadellidae: Iassinae) reveal new gene rearrangements and phylogenetic implications
Source: PeerJ. 2024 Oct 22;12:e18352. doi: 10.7717/peerj.18352 (PMC11505954; doi:10.7717/peerj.18352)
Supplement: Table S3 [file peerj-12-18352-s010.docx]

**Table S3. The relative synonymous codon usage of the PCGs of 11 *Batracomorphus* mitochondrial genomes.**

| ***Batracomorphus allionii*** | | | | | | | | | | | |
| --- | --- | --- | --- | --- | --- | --- | --- | --- | --- | --- | --- |
| **Codon** | **Count** | **RSCU** | **Codon** | **Count** | **RSCU** | **Codon** | **Count** | **RSCU** | **Codon** | **Count** | **RSCU** |
| UUU(F) | 354 | 1.73 | UCU(S) | 92 | 1.95 | UAU(Y) | 151 | 1.63 | UGU(C) | 49 | 1.88 |
| UUC(F) | 55 | 0.27 | UCC(S) | 21 | 0.44 | UAC(Y) | 34 | 0.37 | UGC(C) | 3 | 0.12 |
| UUA(L) | 318 | 4.29 | UCA(S) | 153 | 3.34 | UAA() | 0 | 0 | UGA(W) | 81 | 1.93 |
| UUG(L) | 22 | 0.3 | UCG(S) | 2 | 0.04 | UAG() | 0 | 0 | UGG(W) | 3 | 0.07 |
| CUU(L) | 49 | 0.66 | CCU(P) | 39 | 1.38 | CAU(H) | 46 | 1.46 | CGU(R) | 16 | 1.56 |
| CUC(L) | 4 | 0.05 | CCC(P) | 3 | 0.11 | CAC(H) | 17 | 0.54 | CGC(R) | 1 | 0.1 |
| CUA(L) | 50 | 0.67 | CCA(P) | 69 | 2.44 | CAA(Q) | 42 | 1.75 | CGA(R) | 21 | 2.05 |
| CUG(L) | 2 | 0.03 | CCG(P) | 2 | 0.07 | CAG(Q) | 6 | 0.25 | CGG(R) | 3 | 0.29 |
| AUU(I) | 376 | 1.81 | ACU(T) | 54 | 1.5 | AAU(N) | 163 | 1.65 | AGU(S) | 27 | 0.57 |
| AUC(I) | 39 | 0.19 | ACC(T) | 6 | 0.17 | AAC(N) | 35 | 0.35 | AGC(S) | 1 | 0.02 |
| AUA(M) | 371 | 1.88 | ACA(T) | 84 | 2.33 | AAA(K) | 149 | 1.8 | AGA(S) | 79 | 1.67 |
| AUG(M) | 24 | 0.12 | ACG(T) | 0 | 0 | AAG(K) | 17 | 0.2 | AGG(S) | 3 | 0.06 |
| GUU(V) | 57 | 1.7 | GCU(A) | 23 | 1.42 | GAU(D) | 44 | 1.57 | GGU(G) | 55 | 1.37 |
| GUC(V) | 0 | 0 | GCC(A) | 1 | 0.06 | GAC(D) | 12 | 0.43 | GGC(G) | 2 | 0.05 |
| GUA(V) | 75 | 2.24 | GCA(A) | 40 | 2.46 | GAA(E) | 69 | 1.82 | GGA(G) | 89 | 2.21 |
| GUG(V) | 2 | 0.06 | GCG(A) | 1 | 0.06 | GAG(E) | 7 | 0.18 | GGG(G) | 15 | 0.37 |

| ***Batracomorphus chlorophana*** | | | | | | | | | | | |
| --- | --- | --- | --- | --- | --- | --- | --- | --- | --- | --- | --- |
| **Codon** | **Count** | **RSCU** | **Codon** | **Count** | **RSCU** | **Codon** | **Count** | **RSCU** | **Codon** | **Count** | **RSCU** |
| UUU(F) | 388 | 1.78 | UCU(S) | 115 | 2.44 | UAU(Y) | 162 | 1.71 | UGU(C) | 52 | 1.79 |
| UUC(F) | 49 | 0.22 | UCC(S) | 18 | 0.38 | UAC(Y) | 27 | 0.29 | UGC(C) | 6 | 0.21 |
| UUA(L) | 316 | 4.37 | UCA(S) | 137 | 2.93 | UAA() | 0 | 0 | UGA(W) | 77 | 1.79 |
| UUG(L) | 30 | 0.41 | UCG(S) | 5 | 0.11 | UAG() | 0 | 0 | UGG(W) | 9 | 0.21 |
| CUU(L) | 44 | 0.61 | CCU(P) | 43 | 1.55 | CAU(H) | 48 | 1.6 | CGU(R) | 15 | 1.4 |
| CUC(L) | 3 | 0.04 | CCC(P) | 6 | 0.22 | CAC(H) | 12 | 0.4 | CGC(R) | 1 | 0.09 |
| CUA(L) | 39 | 0.54 | CCA(P) | 61 | 2.2 | CAA(Q) | 41 | 1.71 | CGA(R) | 24 | 2.23 |
| CUG(L) | 2 | 0.03 | CCG(P) | 1 | 0.04 | CAG(Q) | 7 | 0.29 | CGG(R) | 3 | 0.28 |
| AUU(I) | 339 | 1.74 | ACU(T) | 48 | 1.33 | AAU(N) | 171 | 1.64 | AGU(S) | 21 | 0.45 |
| AUC(I) | 50 | 0.26 | ACC(T) | 7 | 0.19 | AAC(N) | 38 | 0.36 | AGC(S) | 3 | 0.06 |
| AUA(M) | 362 | 1.87 | ACA(T) | 86 | 2.39 | AAA(K) | 136 | 1.74 | AGA(S) | 74 | 1.57 |
| AUG(M) | 26 | 0.13 | ACG(T) | 3 | 0.08 | AAG(K) | 20 | 0.26 | AGG(S) | 3 | 0.06 |
| GUU(V) | 65 | 1.97 | GCU(A) | 24 | 1.6 | GAU(D) | 37 | 1.42 | GGU(G) | 66 | 1.56 |
| GUC(V) | 4 | 0.12 | GCC(A) | 3 | 0.2 | GAC(D) | 15 | 0.58 | GGC(G) | 1 | 0.02 |
| GUA(V) | 56 | 1.7 | GCA(A) | 33 | 2.2 | GAA(E) | 71 | 1.75 | GGA(G) | 81 | 1.92 |
| GUG(V) | 7 | 0.21 | GCG(A) | 0 | 0 | GAG(E) | 10 | 0.25 | GGG(G) | 21 | 0.5 |

| ***Batracomorphus cornutus*** | | | | | | | | | | | |
| --- | --- | --- | --- | --- | --- | --- | --- | --- | --- | --- | --- |
| **Codon** | **Count** | **RSCU** | **Codon** | **Count** | **RSCU** | **Codon** | **Count** | **RSCU** | **Codon** | **Count** | **RSCU** |
| UUU(F) | 391 | 1.83 | UCU(S) | 128 | 2.7 | UAU(Y) | 168 | 1.8 | UGU(C) | 49 | 1.75 |
| UUC(F) | 37 | 0.17 | UCC(S) | 15 | 0.32 | UAC(Y) | 19 | 0.2 | UGC(C) | 7 | 0.25 |
| UUA(L) | 346 | 4.57 | UCA(S) | 137 | 2.89 | UAA() | 0 | 0 | UGA(W) | 82 | 1.91 |
| UUG(L) | 24 | 0.32 | UCG(S) | 1 | 0.02 | UAG() | 0 | 0 | UGG(W) | 4 | 0.09 |
| CUU(L) | 41 | 0.54 | CCU(P) | 53 | 1.93 | CAU(H) | 46 | 1.53 | CGU(R) | 13 | 1.24 |
| CUC(L) | 3 | 0.04 | CCC(P) | 6 | 0.22 | CAC(H) | 14 | 0.47 | CGC(R) | 2 | 0.19 |
| CUA(L) | 40 | 0.53 | CCA(P) | 51 | 1.85 | CAA(Q) | 44 | 1.91 | CGA(R) | 26 | 2.48 |
| CUG(L) | 0 | 0 | CCG(P) | 0 | 0 | CAG(Q) | 2 | 0.09 | CGG(R) | 1 | 0.1 |
| AUU(I) | 360 | 1.84 | ACU(T) | 46 | 1.3 | AAU(N) | 161 | 1.61 | AGU(S) | 19 | 0.4 |
| AUC(I) | 32 | 0.16 | ACC(T) | 11 | 0.31 | AAC(N) | 39 | 0.39 | AGC(S) | 2 | 0.04 |
| AUA(M) | 361 | 1.84 | ACA(T) | 83 | 2.34 | AAA(K) | 140 | 1.77 | AGA(S) | 73 | 1.54 |
| AUG(M) | 32 | 0.16 | ACG(T) | 2 | 0.06 | AAG(K) | 18 | 0.23 | AGG(S) | 4 | 0.08 |
| GUU(V) | 54 | 1.74 | GCU(A) | 22 | 1.49 | GAU(D) | 43 | 1.62 | GGU(G) | 61 | 1.42 |
| GUC(V) | 4 | 0.13 | GCC(A) | 0 | 0 | GAC(D) | 10 | 0.38 | GGC(G) | 1 | 0.02 |
| GUA(V) | 61 | 1.97 | GCA(A) | 37 | 2.51 | GAA(E) | 73 | 1.82 | GGA(G) | 97 | 2.26 |
| GUG(V) | 5 | 0.16 | GCG(A) | 0 | 0 | GAG(E) | 7 | 0.17 | GGG(G) | 13 | 0.3 |

| ***Batracomorphus curvatus*** | | | | | | | | | | | |
| --- | --- | --- | --- | --- | --- | --- | --- | --- | --- | --- | --- |
| **Codon** | **Count** | **RSCU** | **Codon** | **Count** | **RSCU** | **Codon** | **Count** | **RSCU** | **Codon** | **Count** | **RSCU** |
| UUU(F) | 391 | 1.83 | UCU(S) | 128 | 2.7 | UAU(Y) | 168 | 1.8 | UGU(C) | 49 | 1.75 |
| UUC(F) | 37 | 0.17 | UCC(S) | 15 | 0.32 | UAC(Y) | 19 | 0.2 | UGC(C) | 7 | 0.25 |
| UUA(L) | 346 | 4.57 | UCA(S) | 137 | 2.89 | UAA() | 0 | 0 | UGA(W) | 82 | 1.91 |
| UUG(L) | 24 | 0.32 | UCG(S) | 1 | 0.02 | UAG() | 0 | 0 | UGG(W) | 4 | 0.09 |
| CUU(L) | 41 | 0.54 | CCU(P) | 53 | 1.93 | CAU(H) | 46 | 1.53 | CGU(R) | 13 | 1.24 |
| CUC(L) | 3 | 0.04 | CCC(P) | 6 | 0.22 | CAC(H) | 14 | 0.47 | CGC(R) | 2 | 0.19 |
| CUA(L) | 40 | 0.53 | CCA(P) | 51 | 1.85 | CAA(Q) | 44 | 1.91 | CGA(R) | 26 | 2.48 |
| CUG(L) | 0 | 0 | CCG(P) | 0 | 0 | CAG(Q) | 2 | 0.09 | CGG(R) | 1 | 0.1 |
| AUU(I) | 360 | 1.84 | ACU(T) | 46 | 1.3 | AAU(N) | 161 | 1.61 | AGU(S) | 19 | 0.4 |
| AUC(I) | 32 | 0.16 | ACC(T) | 11 | 0.31 | AAC(N) | 39 | 0.39 | AGC(S) | 2 | 0.04 |
| AUA(M) | 361 | 1.84 | ACA(T) | 83 | 2.34 | AAA(K) | 140 | 1.77 | AGA(S) | 73 | 1.54 |
| AUG(M) | 32 | 0.16 | ACG(T) | 2 | 0.06 | AAG(K) | 18 | 0.23 | AGG(S) | 4 | 0.08 |
| GUU(V) | 54 | 1.74 | GCU(A) | 22 | 1.49 | GAU(D) | 43 | 1.62 | GGU(G) | 61 | 1.42 |
| GUC(V) | 4 | 0.13 | GCC(A) | 0 | 0 | GAC(D) | 10 | 0.38 | GGC(G) | 1 | 0.02 |
| GUA(V) | 61 | 1.97 | GCA(A) | 37 | 2.51 | GAA(E) | 73 | 1.82 | GGA(G) | 97 | 2.26 |
| GUG(V) | 5 | 0.16 | GCG(A) | 0 | 0 | GAG(E) | 7 | 0.17 | GGG(G) | 13 | 0.3 |

| ***Batracomorphus extentus*** | | | | | | | | | | | |
| --- | --- | --- | --- | --- | --- | --- | --- | --- | --- | --- | --- |
| **Codon** | **Count** | **RSCU** | **Codon** | **Count** | **RSCU** | **Codon** | **Count** | **RSCU** | **Codon** | **Count** | **RSCU** |
| UUU(F) | 357 | 1.7 | UCU(S) | 103 | 2.14 | UAU(Y) | 156 | 1.64 | UGU(C) | 50 | 1.85 |
| UUC(F) | 64 | 0.3 | UCC(S) | 10 | 0.21 | UAC(Y) | 34 | 0.36 | UGC(C) | 4 | 0.15 |
| UUA(L) | 286 | 4.1 | UCA(S) | 156 | 3.34 | UAA() | 0 | 0 | UGA(W) | 78 | 1.84 |
| UUG(L) | 34 | 0.49 | UCG(S) | 1 | 0.02 | UAG() | 0 | 0 | UGG(W) | 7 | 0.16 |
| CUU(L) | 47 | 0.67 | CCU(P) | 41 | 1.52 | CAU(H) | 45 | 1.5 | CGU(R) | 18 | 1.76 |
| CUC(L) | 9 | 0.13 | CCC(P) | 3 | 0.11 | CAC(H) | 15 | 0.5 | CGC(R) | 0 | 0 |
| CUA(L) | 41 | 0.59 | CCA(P) | 63 | 2.33 | CAA(Q) | 48 | 1.85 | CGA(R) | 21 | 2.05 |
| CUG(L) | 2 | 0.03 | CCG(P) | 1 | 0.04 | CAG(Q) | 4 | 0.15 | CGG(R) | 2 | 0.2 |
| AUU(I) | 340 | 1.77 | ACU(T) | 46 | 1.23 | AAU(N) | 153 | 1.54 | AGU(S) | 23 | 0.48 |
| AUC(I) | 45 | 0.23 | ACC(T) | 9 | 0.24 | AAC(N) | 46 | 0.46 | AGC(S) | 2 | 0.04 |
| AUA(M) | 374 | 1.78 | ACA(T) | 91 | 2.44 | AAA(K) | 142 | 1.79 | AGA(S) | 83 | 1.72 |
| AUG(M) | 46 | 0.22 | ACG(T) | 3 | 0.08 | AAG(K) | 17 | 0.21 | AGG(S) | 7 | 0.15 |
| GUU(V) | 68 | 1.89 | GCU(A) | 16 | 1.03 | GAU(D) | 42 | 1.47 | GGU(G) | 64 | 1.57 |
| GUC(V) | 3 | 0.08 | GCC(A) | 6 | 0.39 | GAC(D) | 15 | 0.53 | GGC(G) | 7 | 0.17 |
| GUA(V) | 65 | 1.81 | GCA(A) | 39 | 2.52 | GAA(E) | 62 | 1.7 | GGA(G) | 75 | 1.84 |
| GUG(V) | 8 | 0.22 | GCG(A) | 1 | 0.06 | GAG(E) | 11 | 0.3 | GGG(G) | 17 | 0.42 |
|  | | | | | | | | | | | |
| ***Batracomorphus fuscomaculatus*** | | | | | | | | | | | |
| **Codon** | **Count** | **RSCU** | **Codon** | **Count** | **RSCU** | **Codon** | **Count** | **RSCU** | **Codon** | **Count** | **RSCU** |
| UUU(F) | 381 | 1.81 | UCU(S) | 119 | 2.54 | UAU(Y) | 165 | 1.75 | UGU(C) | 51 | 1.82 |
| UUC(F) | 39 | 0.19 | UCC(S) | 11 | 0.23 | UAC(Y) | 24 | 0.25 | UGC(C) | 5 | 0.18 |
| UUA(L) | 344 | 4.54 | UCA(S) | 145 | 3.09 | UAA() | 0 | 0 | UGA(W) | 78 | 1.81 |
| UUG(L) | 22 | 0.29 | UCG(S) | 5 | 0.11 | UAG() | 0 | 0 | UGG(W) | 8 | 0.19 |
| CUU(L) | 47 | 0.62 | CCU(P) | 43 | 1.55 | CAU(H) | 41 | 1.37 | CGU(R) | 17 | 1.58 |
| CUC(L) | 2 | 0.03 | CCC(P) | 2 | 0.07 | CAC(H) | 19 | 0.63 | CGC(R) | 1 | 0.09 |
| CUA(L) | 38 | 0.5 | CCA(P) | 62 | 2.23 | CAA(Q) | 43 | 1.83 | CGA(R) | 23 | 2.14 |
| CUG(L) | 2 | 0.03 | CCG(P) | 4 | 0.14 | CAG(Q) | 4 | 0.17 | CGG(R) | 2 | 0.19 |
| AUU(I) | 363 | 1.8 | ACU(T) | 41 | 1.12 | AAU(N) | 160 | 1.63 | AGU(S) | 19 | 0.41 |
| AUC(I) | 41 | 0.2 | ACC(T) | 10 | 0.27 | AAC(N) | 36 | 0.37 | AGC(S) | 0 | 0 |
| AUA(M) | 361 | 1.89 | ACA(T) | 96 | 2.61 | AAA(K) | 146 | 1.8 | AGA(S) | 74 | 1.58 |
| AUG(M) | 22 | 0.11 | ACG(T) | 0 | 0 | AAG(K) | 16 | 0.2 | AGG(S) | 2 | 0.04 |
| GUU(V) | 64 | 2.08 | GCU(A) | 26 | 1.79 | GAU(D) | 45 | 1.7 | GGU(G) | 66 | 1.56 |
| GUC(V) | 1 | 0.03 | GCC(A) | 0 | 0 | GAC(D) | 8 | 0.3 | GGC(G) | 2 | 0.05 |
| GUA(V) | 56 | 1.82 | GCA(A) | 31 | 2.14 | GAA(E) | 77 | 1.88 | GGA(G) | 84 | 1.99 |
| GUG(V) | 2 | 0.07 | GCG(A) | 1 | 0.07 | GAG(E) | 5 | 0.12 | GGG(G) | 17 | 0.4 |

| ***Batracomorphus lineatus*** | | | | | | | | | | | |
| --- | --- | --- | --- | --- | --- | --- | --- | --- | --- | --- | --- |
| **Codon** | **Count** | **RSCU** | **Codon** | **Count** | **RSCU** | **Codon** | **Count** | **RSCU** | **Codon** | **Count** | **RSCU** |
| UUU(F) | 356 | 1.7 | UCU(S) | 93 | 1.95 | UAU(Y) | 143 | 1.51 | UGU(C) | 50 | 1.79 |
| UUC(F) | 62 | 0.3 | UCC(S) | 16 | 0.34 | UAC(Y) | 47 | 0.49 | UGC(C) | 6 | 0.21 |
| UUA(L) | 285 | 3.9 | UCA(S) | 149 | 3.23 | UAA() | 0 | 0 | UGA(W) | 73 | 1.74 |
| UUG(L) | 32 | 0.44 | UCG(S) | 7 | 0.15 | UAG() | 0 | 0 | UGG(W) | 11 | 0.26 |
| CUU(L) | 45 | 0.62 | CCU(P) | 37 | 1.33 | CAU(H) | 45 | 1.38 | CGU(R) | 18 | 1.76 |
| CUC(L) | 6 | 0.08 | CCC(P) | 6 | 0.22 | CAC(H) | 20 | 0.62 | CGC(R) | 0 | 0 |
| CUA(L) | 63 | 0.86 | CCA(P) | 64 | 2.31 | CAA(Q) | 43 | 1.76 | CGA(R) | 20 | 1.95 |
| CUG(L) | 8 | 0.11 | CCG(P) | 4 | 0.14 | CAG(Q) | 6 | 0.24 | CGG(R) | 3 | 0.29 |
| AUU(I) | 330 | 1.71 | ACU(T) | 44 | 1.19 | AAU(N) | 154 | 1.61 | AGU(S) | 26 | 0.55 |
| AUC(I) | 55 | 0.29 | ACC(T) | 9 | 0.24 | AAC(N) | 37 | 0.39 | AGC(S) | 0 | 0 |
| AUA(M) | 369 | 1.78 | ACA(T) | 92 | 2.49 | AAA(K) | 144 | 1.78 | AGA(S) | 80 | 1.68 |
| AUG(M) | 45 | 0.22 | ACG(T) | 3 | 0.08 | AAG(K) | 18 | 0.22 | AGG(S) | 10 | 0.21 |
| GUU(V) | 57 | 1.66 | GCU(A) | 21 | 1.29 | GAU(D) | 42 | 1.53 | GGU(G) | 67 | 1.69 |
| GUC(V) | 6 | 0.18 | GCC(A) | 4 | 0.25 | GAC(D) | 13 | 0.47 | GGC(G) | 3 | 0.08 |
| GUA(V) | 69 | 2.01 | GCA(A) | 35 | 2.15 | GAA(E) | 63 | 1.64 | GGA(G) | 72 | 1.81 |
| GUG(V) | 5 | 0.15 | GCG(A) | 5 | 0.31 | GAG(E) | 14 | 0.36 | GGG(G) | 17 | 0.43 |

| ***Batracomorphus matsumurai*** | | | | | | | | | | | |
| --- | --- | --- | --- | --- | --- | --- | --- | --- | --- | --- | --- |
| **Codon** | **Count** | **RSCU** | **Codon** | **Count** | **RSCU** | **Codon** | **Count** | **RSCU** | **Codon** | **Count** | **RSCU** |
| UUU(F) | 388 | 1.79 | UCU(S) | 128 | 2.7 | UAU(Y) | 156 | 1.64 | UGU(C) | 40 | 1.82 |
| UUC(F) | 45 | 0.21 | UCC(S) | 14 | 0.3 | UAC(Y) | 34 | 0.36 | UGC(C) | 4 | 0.18 |
| UUA(L) | 322 | 4.43 | UCA(S) | 126 | 2.66 | UAA() | 0 | 0 | UGA(W) | 75 | 1.83 |
| UUG(L) | 29 | 0.4 | UCG(S) | 5 | 0.11 | UAG() | 0 | 0 | UGG(W) | 7 | 0.17 |
| CUU(L) | 46 | 0.63 | CCU(P) | 49 | 1.81 | CAU(H) | 41 | 1.41 | CGU(R) | 16 | 1.45 |
| CUC(L) | 1 | 0.01 | CCC(P) | 5 | 0.19 | CAC(H) | 17 | 0.59 | CGC(R) | 0 | 0 |
| CUA(L) | 35 | 0.48 | CCA(P) | 51 | 1.89 | CAA(Q) | 48 | 1.92 | CGA(R) | 23 | 2.09 |
| CUG(L) | 3 | 0.04 | CCG(P) | 3 | 0.11 | CAG(Q) | 2 | 0.08 | CGG(R) | 5 | 0.45 |
| AUU(I) | 379 | 1.82 | ACU(T) | 49 | 1.56 | AAU(N) | 171 | 1.69 | AGU(S) | 27 | 0.57 |
| AUC(I) | 38 | 0.18 | ACC(T) | 11 | 0.35 | AAC(N) | 31 | 0.31 | AGC(S) | 2 | 0.04 |
| AUA(M) | 367 | 1.87 | ACA(T) | 64 | 2.03 | AAA(K) | 144 | 1.83 | AGA(S) | 75 | 1.58 |
| AUG(M) | 26 | 0.13 | ACG(T) | 2 | 0.06 | AAG(K) | 13 | 0.17 | AGG(S) | 2 | 0.04 |
| GUU(V) | 78 | 2.35 | GCU(A) | 28 | 1.44 | GAU(D) | 36 | 1.47 | GGU(G) | 54 | 1.31 |
| GUC(V) | 3 | 0.09 | GCC(A) | 5 | 0.26 | GAC(D) | 13 | 0.53 | GGC(G) | 5 | 0.12 |
| GUA(V) | 49 | 1.47 | GCA(A) | 42 | 2.15 | GAA(E) | 73 | 1.82 | GGA(G) | 86 | 2.08 |
| GUG(V) | 3 | 0.09 | GCG(A) | 3 | 0.15 | GAG(E) | 7 | 0.17 | GGG(G) | 20 | 0.48 |

| ***Batracomorphus nigromarginattus*** | | | | | | | | | | | |
| --- | --- | --- | --- | --- | --- | --- | --- | --- | --- | --- | --- |
| **Codon** | **Count** | **RSCU** | **Codon** | **Count** | **RSCU** | **Codon** | **Count** | **RSCU** | **Codon** | **Count** | **RSCU** |
| UUU(F) | 364 | 1.77 | UCU(S) | 90 | 1.94 | UAU(Y) | 163 | 1.72 | UGU(C) | 51 | 1.92 |
| UUC(F) | 48 | 0.23 | UCC(S) | 9 | 0.19 | UAC(Y) | 26 | 0.28 | UGC(C) | 2 | 0.08 |
| UUA(L) | 305 | 4.34 | UCA(S) | 157 | 3.49 | UAA() | 0 | 0 | UGA(W) | 80 | 1.88 |
| UUG(L) | 27 | 0.38 | UCG(S) | 4 | 0.09 | UAG() | 0 | 0 | UGG(W) | 5 | 0.12 |
| CUU(L) | 49 | 0.7 | CCU(P) | 40 | 1.43 | CAU(H) | 44 | 1.44 | CGU(R) | 15 | 1.43 |
| CUC(L) | 3 | 0.04 | CCC(P) | 8 | 0.29 | CAC(H) | 17 | 0.56 | CGC(R) | 0 | 0 |
| CUA(L) | 37 | 0.53 | CCA(P) | 63 | 2.25 | CAA(Q) | 49 | 1.92 | CGA(R) | 25 | 2.38 |
| CUG(L) | 1 | 0.01 | CCG(P) | 1 | 0.04 | CAG(Q) | 2 | 0.08 | CGG(R) | 2 | 0.19 |
| AUU(I) | 371 | 1.82 | ACU(T) | 53 | 1.45 | AAU(N) | 164 | 1.66 | AGU(S) | 28 | 0.6 |
| AUC(I) | 37 | 0.18 | ACC(T) | 4 | 0.11 | AAC(N) | 34 | 0.34 | AGC(S) | 0 | 0 |
| AUA(M) | 410 | 1.89 | ACA(T) | 87 | 2.38 | AAA(K) | 149 | 1.8 | AGA(S) | 76 | 1.64 |
| AUG(M) | 23 | 0.11 | ACG(T) | 2 | 0.05 | AAG(K) | 17 | 0.2 | AGG(S) | 7 | 0.15 |
| GUU(V) | 62 | 1.91 | GCU(A) | 26 | 1.7 | GAU(D) | 45 | 1.73 | GGU(G) | 60 | 1.54 |
| GUC(V) | 1 | 0.03 | GCC(A) | 1 | 0.07 | GAC(D) | 7 | 0.27 | GGC(G) | 2 | 0.05 |
| GUA(V) | 65 | 2 | GCA(A) | 33 | 2.16 | GAA(E) | 72 | 1.85 | GGA(G) | 83 | 2.13 |
| GUG(V) | 2 | 0.06 | GCG(A) | 1 | 0.07 | GAG(E) | 6 | 0.15 | GGG(G) | 11 | 0.28 |

| ***Batracomorphus notatus*** | | | | | | | | | | | |
| --- | --- | --- | --- | --- | --- | --- | --- | --- | --- | --- | --- |
| **Codon** | **Count** | **RSCU** | **Codon** | **Count** | **RSCU** | **Codon** | **Count** | **RSCU** | **Codon** | **Count** | **RSCU** |
| UUU(F) | 377 | 1.8 | UCU(S) | 122 | 2.62 | UAU(Y) | 173 | 1.81 | UGU(C) | 50 | 1.79 |
| UUC(F) | 42 | 0.2 | UCC(S) | 13 | 0.28 | UAC(Y) | 18 | 0.19 | UGC(C) | 6 | 0.21 |
| UUA(L) | 318 | 4.29 | UCA(S) | 139 | 2.98 | UAA() | 0 | 0 | UGA(W) | 73 | 1.7 |
| UUG(L) | 40 | 0.54 | UCG(S) | 5 | 0.11 | UAG() | 0 | 0 | UGG(W) | 13 | 0.3 |
| CUU(L) | 44 | 0.59 | CCU(P) | 48 | 1.73 | CAU(H) | 41 | 1.37 | CGU(R) | 18 | 1.71 |
| CUC(L) | 3 | 0.04 | CCC(P) | 5 | 0.18 | CAC(H) | 19 | 0.63 | CGC(R) | 1 | 0.1 |
| CUA(L) | 39 | 0.53 | CCA(P) | 57 | 2.05 | CAA(Q) | 45 | 1.88 | CGA(R) | 22 | 2.1 |
| CUG(L) | 1 | 0.01 | CCG(P) | 1 | 0.04 | CAG(Q) | 3 | 0.13 | CGG(R) | 1 | 0.1 |
| AUU(I) | 358 | 1.79 | ACU(T) | 43 | 1.21 | AAU(N) | 167 | 1.65 | AGU(S) | 15 | 0.32 |
| AUC(I) | 43 | 0.21 | ACC(T) | 5 | 0.14 | AAC(N) | 36 | 0.35 | AGC(S) | 4 | 0.09 |
| AUA(M) | 364 | 1.84 | ACA(T) | 93 | 2.62 | AAA(K) | 148 | 1.85 | AGA(S) | 73 | 1.57 |
| AUG(M) | 31 | 0.16 | ACG(T) | 1 | 0.03 | AAG(K) | 12 | 0.15 | AGG(S) | 2 | 0.04 |
| GUU(V) | 70 | 2.2 | GCU(A) | 26 | 1.82 | GAU(D) | 49 | 1.81 | GGU(G) | 60 | 1.4 |
| GUC(V) | 0 | 0 | GCC(A) | 1 | 0.07 | GAC(D) | 5 | 0.19 | GGC(G) | 1 | 0.02 |
| GUA(V) | 56 | 1.76 | GCA(A) | 29 | 2.04 | GAA(E) | 75 | 1.83 | GGA(G) | 84 | 1.96 |
| GUG(V) | 1 | 0.03 | GCG(A) | 1 | 0.07 | GAG(E) | 7 | 0.17 | GGG(G) | 26 | 0.61 |

| ***Batracomorphus rinkihonis*** | | | | | | | | | | | |
| --- | --- | --- | --- | --- | --- | --- | --- | --- | --- | --- | --- |
| **Codon** | **Count** | **RSCU** | **Codon** | **Count** | **RSCU** | **Codon** | **Count** | **RSCU** | **Codon** | **Count** | **RSCU** |
| UUU(F) | 378 | 1.81 | UCU(S) | 116 | 2.45 | UAU(Y) | 171 | 1.76 | UGU(C) | 50 | 1.96 |
| UUC(F) | 40 | 0.19 | UCC(S) | 13 | 0.27 | UAC(Y) | 23 | 0.24 | UGC(C) | 1 | 0.04 |
| UUA(L) | 320 | 4.4 | UCA(S) | 151 | 3.29 | UAA() | 0 | 0 | UGA(W) | 79 | 1.82 |
| UUG(L) | 36 | 0.5 | UCG(S) | 3 | 0.06 | UAG() | 0 | 0 | UGG(W) | 8 | 0.18 |
| CUU(L) | 40 | 0.55 | CCU(P) | 52 | 1.87 | CAU(H) | 52 | 1.73 | CGU(R) | 16 | 1.49 |
| CUC(L) | 2 | 0.03 | CCC(P) | 3 | 0.11 | CAC(H) | 8 | 0.27 | CGC(R) | 1 | 0.09 |
| CUA(L) | 35 | 0.48 | CCA(P) | 55 | 1.98 | CAA(Q) | 41 | 1.74 | CGA(R) | 25 | 2.33 |
| CUG(L) | 3 | 0.04 | CCG(P) | 1 | 0.04 | CAG(Q) | 6 | 0.26 | CGG(R) | 1 | 0.09 |
| AUU(I) | 372 | 1.87 | ACU(T) | 46 | 1.25 | AAU(N) | 172 | 1.68 | AGU(S) | 26 | 0.55 |
| AUC(I) | 25 | 0.13 | ACC(T) | 11 | 0.3 | AAC(N) | 33 | 0.32 | AGC(S) | 0 | 0 |
| AUA(M) | 379 | 1.87 | ACA(T) | 89 | 2.42 | AAA(K) | 140 | 1.77 | AGA(S) | 68 | 1.44 |
| AUG(M) | 27 | 0.13 | ACG(T) | 1 | 0.03 | AAG(K) | 18 | 0.23 | AGG(S) | 2 | 0.04 |
| GUU(V) | 60 | 1.98 | GCU(A) | 21 | 1.45 | GAU(D) | 45 | 1.73 | GGU(G) | 64 | 1.51 |
| GUC(V) | 2 | 0.07 | GCC(A) | 3 | 0.21 | GAC(D) | 7 | 0.27 | GGC(G) | 0 | 0 |
| GUA(V) | 55 | 1.82 | GCA(A) | 32 | 2.21 | GAA(E) | 72 | 1.78 | GGA(G) | 89 | 2.09 |
| GUG(V) | 4 | 0.13 | GCG(A) | 2 | 0.14 | GAG(E) | 9 | 0.22 | GGG(G) | 17 | 0.4 |
