# Supplementary material for: The mitochondrial genome sequences of eleven leafhopper species of Batracomorphus (Hemiptera: Cicadellidae: Iassinae) reveal new gene rearrangements and phylogenetic implications
Source: PeerJ. 2024 Oct 22;12:e18352. doi: 10.7717/peerj.18352 (PMC11505954; doi:10.7717/peerj.18352)
Supplement: Table S4–S14 [file peerj-12-18352-s011.docx]

**Table S4 Mitochondrial genome organization of *Batracomorphus allionii*.**

| **Gene** | **Strand** | **Position** | **Size (bp)** | **Start / Stop codon** | **IN** |
| --- | --- | --- | --- | --- | --- |
| *trnQ* | N | 1-69 | 69 |  | 47 |
| *trnI* | J | 117-180 | 64 |  | 0 |
| *trnM* | J | 181-248 | 68 |  | 0 |
| *nad2* | J | 249-1,217 | 969 | ATA/TAA | -2 |
| *trnW* | J | 1,216-1,277 | 62 |  | -8 |
| *trnC* | N | 1,270-1,330 | 61 |  | 8 |
| *trnY* | N | 1,339-1,403 | 65 |  | 10 |
| *cox1* | J | 1,414-2,952 | 1,539 | ATG/ TAA | -5 |
| *trnL*2^(UUR^*^)^* | J | 2,948-3,013 | 66 |  | 0 |
| *cox2* | J | 3,014-3,683 | 670 | ATA/T-- | 0 |
| *trnK* | J | 3,684-3,755 | 72 |  | -1 |
| *trnD* | J | 3,755-3,814 | 60 |  | -3 |
| *atp8* | J | 3,812-3,964 | 153 | TTG/TAA | -7 |
| *atp6* | J | 3,958-4,593 | 636 | ATA/TAA | 0 |
| *cox3* | J | 4,594-5,373 | 780 | ATG/T-- | 3 |
| *trnG* | J | 5,377-5,435 | 59 |  | 0 |
| *nad3* | J | 5,436-5,789 | 354 | ATT/TAG | -2 |
| *trnA* | J | 5,788-5,847 | 60 |  | -2 |
| *trnR* | J | 5,846-5,912 | 67 |  | -2 |
| *trnN* | J | 5,911-5,974 | 64 |  | -1 |
| *trnS*1^(AGN)^ | J | 5,974-6,040 | 67 |  | 6 |
| *trnE* | J | 6,047-6,109 | 63 |  | 0 |
| *trnF* | N | 6,110-6,169 | 60 |  | 0 |
| *nad5* | N | 6,170-7,847 | 1,678 | TTG/T-- | -6 |
| *trnH* | N | 7,842-7,905 | 64 |  | 4 |
| *nad4* | N | 7,910-9,211 | 1,302 | ATG/TAA | -7 |
| *nad4L* | N | 9,205-9,480 | 276 | ATG/TAG | 2 |
| *trnT* | J | 9,483-9,543 | 61 |  | 0 |
| *trnP* | N | 9,544-9,606 | 63 |  | 2 |
| *nad6* | J | 9,609-10,094 | 486 | ATA/TAG | -1 |
| *Cytb* | J | 10,094-11,227 | 1,134 | ATG/TAG | 3 |
| *trnS*2^(UCN)^ | J | 11,231-11,293 | 63 |  | -10 |
| *nad1* | N | 11,284-12,225 | 942 | ATT/TAA | 0 |
| *trnL*1^(CUN)^ | N | 12,226-12,289 | 64 |  | 0 |
| *lrRNA* | N | 12,290-13,455 | 1,166 |  | 0 |
| *trnV* | N | 13,456-13,516 | 61 |  | 0 |
| *srRNA* | N | 13,517-14,224 | 708 |  | 0 |
| CR |  | 14,225-15,025 | 801 |  | 0 |

**Table S5 Mitochondrial genome organization of *Batracomorphus chlorophana***

| **Gene** | **Strand** | **Position** | **Size (bp)** | **Start / Stop codon** | **IN** |
| --- | --- | --- | --- | --- | --- |
| *trnI* | J | 1-63 | 63 |  | -3 |
| *trnQ* | N | 61-129 | 69 |  | -1 |
| *trnM* | J | -197 | 69 |  | 0 |
| *nad2* | J | 198-1,163 | 966 | ATT/TAA | -2 |
| *trnW* | J | 1,162-1,226 | 65 |  | -8 |
| *trnC* | N | 1,219-1,279 | 61 |  | -1 |
| *trnY* | N | 1,279-1,345 | 67 |  | -2 |
| *cox1* | J | 1,344-2,882 | 1,539 | ATG/TAA | -5 |
| *trnL2*^(UUR)^ | J | 2,878-2,944 | 67 |  | 0 |
| *cox2* | J | 2,945-3,611 | 667 | ATT/T-- | 0 |
| *trnK* | J | 3,612-3,683 | 72 |  | 0 |
| *trnD* | J | 3,684-3,745 | 62 |  | -3 |
| *atp8* | J | 3,743-4,892 | 150 | TTG/TAA | -4 |
| *atp6* | J | 3,889-4,524 | 636 | ATA/TAA | 0 |
| *cox3* | J | 4,525-5,302 | 778 | ATG/T-- | 0 |
| *trnG* | J | 5,303-5,361 | 59 |  | -1 |
| *nad3* | J | 5,361-5,714 | 354 | ATT/TAA | -1 |
| *trnA* | J | 5,714-5,774 | 61 |  | -2 |
| *trnR* | J | 5,773-5,837 | 65 |  | -2 |
| *trnN* | J | 5,836-5,898 | 63 |  | -1 |
| *trnS*1^(AGN)^ | J | 5,898-5,964 | 67 |  | 2 |
| *trnE* | J | 5,967-6,026 | 60 |  | 7 |
| *trnF* | N | 6,034-6,099 | 66 |  | -1 |
| *nad5* | N | 6,099-7,775 | 1,677 | TTG/TAG | 0 |
| *trnH* | N | 7,776-7,839 | 64 |  | 6 |
| *nad4* | N | 7,846-9,147 | 1,302 | ATG/TAA | -7 |
| *nad4L* | N | 9,141-9,416 | 276 | ATG/TAA | 2 |
| *trnT* | J | 9,419-9,480 | 62 |  | 0 |
| *trnP* | N | 9,481-9,545 | 65 |  | 2 |
| *nad6* | J | 9,548-10,030 | 483 | ATC/TAA | -1 |
| *Cytb* | J | 10,030-11,163 | 1,134 | ATG/TAG | -2 |
| *trnS2*^(UCN)^ | J | 11,162-11,224 | 63 |  | -10 |
| *nad1* | N | 11,215-12,156 | 942 | ATT/TAA | 0 |
| *trnL*1^(CUN)^ | N | 12,157-12,221 | 65 |  | 0 |
| *lrRNA* | N | 12,222-13,391 | 1,170 |  | 0 |
| *trnV* | N | 13,392-13,456 | 65 |  | 0 |
| *srRNA* | N | 13,457-14,172 | 716 |  | 0 |
| CR |  | 14,173-14,870 | 698 |  | 0 |

**Table S6 Mitochondrial genome organization of *Batracomorphus cornutus***

| **Gene** | **Strand** | **Position** | **Size (bp)** | **Start / Stop codon** | **IN** |
| --- | --- | --- | --- | --- | --- |
| *trnI* | J | 1-62 | 62 |  | -3 |
| *trnQ* | N | 60-128 | 69 |  | -1 |
| *trnM* | J | 128-195 | 68 |  | 0 |
| *nad2* | J | 196-1,161 | 966 | ATT/TAA | -2 |
| *trnW* | J | 1,160-1,224 | 65 |  | -8 |
| *trnC* | N | 1,217-1,277 | 61 |  | 2 |
| *trnY* | N | 1,280-1,347 | 68 |  | 2 |
| *cox1* | J | 1,350-2,888 | 1,539 | ATG/TAA | -5 |
| *trnL2*^(UUR)^ | J | 2,884-2,950 | 67 |  | 0 |
| *cox2* | J | 2,951-3,617 | 667 | ATT/T-- | 0 |
| *trnK* | J | 3,618-3,689 | 72 |  | 2 |
| *trnD* | J | 3,692-3,754 | 63 |  | -3 |
| *atp8* | J | 3,752-3,901 | 150 | TTG/TAA | -4 |
| *atp6* | J | 3,898-4,533 | 636 | ATA/TAA | 1 |
| *cox3* | J | 4,535-5,312 | 778 | ATG/T-- | 0 |
| *trnG* | J | 5,313-5,371 | 59 |  | 0 |
| *nad3* | J | 5,372-5,725 | 354 | ATT/TAA | -2 |
| *trnA* | J | 5,724-5,784 | 61 |  | -1 |
| *trnR* | J | 5,784-5,843 | 60 |  | -1 |
| *trnN* | J | 5,843-5,904 | 62 |  | -1 |
| *trnS*1^(AGN)^ | J | 5,904-5,970 | 67 |  | 2 |
| *trnE* | J | 5,973-6,037 | 65 |  | 4 |
| *trnF* | N | 6,042-6,103 | 62 |  | -1 |
| *nad5* | N | 6,103-7,779 | 1,677 | TTG/TAG | 0 |
| *trnH* | N | 7,780-7,844 | 65 |  | 10 |
| *nad4* | N | 7,855-9,156 | 1,302 | ATG/TAA | -7 |
| *nad4L* | N | 9,150-9,425 | 276 | ATG/TAA | 2 |
| *trnT* | J | 9,428-9,489 | 62 |  | 0 |
| *trnP* | N | 9,490-9,554 | 65 |  | 2 |
| *nad6* | J | 9,557-10,039 | 483 | ATC/TAA | -1 |
| *Cytb* | J | 10,039-11,172 | 1,134 | ATG/TAG | 1 |
| *trnS2*^(UCN)^ | J | 11,174-11,239 | 66 |  | -1 |
| *nad1* | N | 11,239-12,174 | 936 | ATT/TAA | -3 |
| *trnL*1^(CUN)^ | N | 12,172-12,236 | 65 |  | 0 |
| *lrRNA* | N | 12,237-13,413 | 1,177 |  | 0 |
| *trnV* | N | 13,414-13,478 | 65 |  | 0 |
| *srRNA* | N | 13,479-14,189 | 711 |  | 0 |
| CR |  | 14,190-15,021 | 832 |  | 0 |

**Table S7 Mitochondrial genome organization of *Batracomorphus curvatus***

| **Gene** | **Strand** | **Position** | **Size (bp)** | **Start / Stop codon** | **IN** |
| --- | --- | --- | --- | --- | --- |
| *trnI* | J | 1-63 | 63 |  | -3 |
| *trnQ* | N | 61-129 | 69 |  | 0 |
| *trnM* | J | 130-195 | 66 |  | 0 |
| *nad2* | J | 196-1,158 | 963 | ATT/TAA | -1 |
| *trnW* | J | 1,158-1,220 | 63 |  | -8 |
| *trnC* | N | 1,213-2,274 | 62 |  | -1 |
| *trnY* | N | 1,274-1,336 | 63 |  | 4 |
| *cox1* | J | 1,341-2,879 | 1,539 | ATG/TAA | -5 |
| *trnL2*^(UUR)^ | J | 2,875-2,943 | 69 |  | 0 |
| *cox2* | J | 2,944-3,613 | 670 | ATT/T-- | 0 |
| *trnK* | J | 3,614-3,684 | 71 |  | 4 |
| *trnD* | J | 3,689-3,749 | 61 |  | -3 |
| *atp8* | J | 3,747-3,896 | 150 | TTG/TAA | -4 |
| *atp6* | J | 3,893-5,528 | 636 | ATA/TAA | 2 |
| *cox3* | J | 4,531-5,308 | 778 | ATG/T-- | 0 |
| *trnG* | J | 5,309-5,367 | 59 |  | 0 |
| *nad3* | J | 5,368-5,721 | 354 | ATT/TAG | 0 |
| *trnA* | J | 5,722-5,783 | 62 |  | 6 |
| *trnR* | J | 5,790-5,851 | 62 |  | -1 |
| *trnN* | J | 5,851-5,914 | 64 |  | -1 |
| *trnS*1^(AGN)^ | J | 5,914-5,980 | 67 |  | 2 |
| *trnE* | J | 5,983-6,045 | 63 |  | 2 |
| *trnF* | N | 6,048-6,110 | 63 |  | 0 |
| *nad5* | N | 6,111-7,788 | 1,678 | ATT/T-- | -3 |
| *trnH* | N | 7,786-7,846 | 61 |  | 0 |
| *nad4* | N | 7,847-9,146 | 1,300 | ATG/T-- | -7 |
| *nad4L* | N | 9,140-9,412 | 273 | ATG/TAA | 2 |
| *trnT* | J | 9,415-9,478 | 64 |  | 0 |
| *trnP* | N | 9,479-9,543 | 65 |  | 2 |
| *nad6* | J | 9,546-10,031 | 486 | ATA/TAA | -1 |
| *Cytb* | J | 10,031-11,161 | 1,131 | ATG/TAG | -2 |
| *trnS2*^(UCN)^ | J | 11,160-11,222 | 63 |  | -7 |
| *nad1* | N | 11,216-12,157 | 942 | ATT/TAA | -3 |
| *trnL*1^(CUN)^ | N | 12,155-12,221 | 67 |  | 0 |
| *lrRNA* | N | 12,222-13,389 | 1,168 |  | 0 |
| *trnV* | N | 13,390-13,451 | 62 |  | 0 |
| *srRNA* | N | 13,452-14,163 | 712 |  | 0 |
| CR |  | 14,164-15,375 | 1,212 |  | 0 |

**Table S8 Mitochondrial genome organization of *Batracomorphus extentus***

| **Gene** | **Strand** | **Position** | **Size (bp)** | **Start / Stop codon** | **IN** |
| --- | --- | --- | --- | --- | --- |
| *trnQ* | N | 1-69 | 69 |  | 43 |
| *trnI* | J | 113-279 | 67 |  | 0 |
| *trnM* | J | 180-251 | 72 |  | 0 |
| *nad2* | J | 252-1,220 | 969 | ATA/TAA | -2 |
| *trnW* | J | 1,219-1,280 | 62 |  | -8 |
| *trnC* | N | 1,273-2,331 | 59 |  | 2 |
| *trnY* | N | 1,334-1,399 | 66 |  | 24 |
| *cox1* | J | 1,424-2,962 | 1,539 | ATG/TAA | -5 |
| *trnL*2^(UUR)^ | J | 2,958-3,023 | 66 |  | 0 |
| *cox2* | J | 3,024-3,693 | 670 | ATA/T-- | 0 |
| *trnK* | J | 3,694-3,764 | 71 |  | 1 |
| *trnD* | J | 3,766-3,828 | 63 |  | -3 |
| *atp8* | J | 3,826-3,978 | 153 | TTG/TAA | -7 |
| *atp6* | J | 3,972-4,607 | 636 | ATG/TAA | 2 |
| *cox3* | J | 4,610-5,389 | 780 | ATG/TAG | 2 |
| *trnG* | J | 5,392-5,444 | 53 |  | 9 |
| *nad3* | J | 5,454-5,807 | 354 | ATA/TAA | 3 |
| *trnA* | J | 5,811-5,870 | 60 |  | -2 |
| *trnR* | J | 5,869-5,936 | 68 |  | -2 |
| *trnN* | J | 5,935-6,002 | 68 |  | -1 |
| *trnS*1^(AGN)^ | J | 6,002-6,068 | 67 |  | 0 |
| *trnE* | J | 6,069-6,132 | 64 |  | -2 |
| *trnF* | N | 6,131-6,192 | 62 |  | 0 |
| *nad5* | N | 6,193-7,870 | 1,678 | ATT/T-- | -3 |
| *trnH* | N | 7,868-7,926 | 59 |  | 0 |
| *nad4* | N | 7,927-9,228 | 1,302 | ATG/TAA | -7 |
| *nad4L* | N | 9,222-9,497 | 276 | ATG/TAA | 16 |
| *trnT* | J | 9,514-9,574 | 61 |  | 0 |
| *trnP* | N | 9,575-9,638 | 64 |  | 8 |
| *nad6* | J | 9,647-10,129 | 483 | ATT/TAA | -1 |
| *Cytb* | J | 10,129-11,262 | 1,134 | ATG/TAG | -2 |
| *trnS*2^(UCN)^ | J | 11,261-11,323 | 63 |  | -10 |
| *nad1* | N | 11,314-12,255 | 942 | ATT/TAA | 0 |
| *trnL*1^(CUN)^ | N | 12,256-12,319 | 64 |  | 0 |
| *lrRNA* | N | 12,320-13,484 | 1,165 |  | 0 |
| *trnV* | N | 13,485-13,545 | 61 |  | 0 |
| *srRNA* | N | 13,546-14,253 | 708 |  | 0 |
| CR |  | 14,254-16,139 | 886 |  | 0 |

**Table S9 Mitochondrial genome organization of *Batracomorphus fuscomaculatus***

| **Gene** | **Strand** | **Position** | **Size (bp)** | **Start / Stop codon** | **IN** |
| --- | --- | --- | --- | --- | --- |
| *trnI* | J | 1-62 | 62 |  | -3 |
| *trnQ* | N | 60-128 | 69 |  | -1 |
| *trnM* | J | 128-195 | 68 |  | 0 |
| *nad2* | J | 196-1,161 | 966 | ATT/TAA | -2 |
| *trnW* | J | 1,160-1,224 | 65 |  | -8 |
| *trnC* | N | 1,217-1,279 | 63 |  | -1 |
| *trnY* | N | 1,279-1,345 | 67 |  | -2 |
| *cox1* | J | 1,344-2,882 | 1,539 | ATG/TAA | -5 |
| *trnL2*^(UUR)^ | J | 2,878-2,943 | 66 |  | 0 |
| *cox2* | J | 2,944-3,610 | 667 | ATT/T-- | 0 |
| *trnK* | J | 3,611-3,682 | 72 |  | 0 |
| *trnD* | J | 3,683-4,744 | 62 |  | -3 |
| *atp8* | J | 3,742-3,891 | 150 | TTG/TAA | -4 |
| *atp6* | J | 3,888-4,523 | 636 | ATA/TAA | 0 |
| *cox3* | J | 4,524-5,301 | 778 | ATG/T-- | 0 |
| *trnG* | J | 5,302-5,360 | 59 |  | 0 |
| *nad3* | J | 5,361-5,714 | 354 | ATT/TAA | -1 |
| *trnA* | J | 5,714-5,774 | 61 |  | -1 |
| *trnR* | J | 5,774-5,835 | 62 |  | -1 |
| *trnN* | J | 5,835-5,897 | 63 |  | -1 |
| *trnS*1^(AGN)^ | J | 5,897-5,963 | 67 |  | 5 |
| *trnE* | J | 5,969-6,029 | 61 |  | 5 |
| *trnF* | N | 6,035-6,097 | 63 |  | -1 |
| *nad5* | N | 6,097-7,773 | 1,677 | TTG/TAG | 0 |
| *trnH* | N | 7,774-7,839 | 66 |  | 5 |
| *nad4* | N | 7,845-9,146 | 1,302 | ATG/TAG | -7 |
| *nad4L* | N | 9,140-9,415 | 276 | ATG/TAA | 22 |
| *trnT* | J | 9,438-9,499 | 62 |  | 0 |
| *trnP* | N | 9,500-9,564 | 65 |  | 2 |
| *nad6* | J | 9,567-10,049 | 483 | ATT/TAA | -1 |
| *Cytb* | J | 10,049-11,182 | 1,134 | ATG/TAA | 0 |
| *trnS2*^(UCN)^ | J | 11,183-11,244 | 62 |  | -1 |
| *nad1* | N | 11,244-12,179 | 936 | ATA/TAA | -3 |
| *trnL*1^(CUN)^ | N | 12,177-12,242 | 66 |  | 0 |
| *lrRNA* | N | 12,243-13,414 | 1,172 |  | 0 |
| *trnV* | N | 13,415-13,478 | 64 |  | 0 |
| *srRNA* | N | 13,479-14,178 | 700 |  | 0 |
| CR |  | 14,179-14,915 | 737 |  | 0 |

**Table S10 Mitochondrial genome organization of *Batracomorphus lineatus***

| **Gene** | **Strand** | **Position** | **Size (bp)** | **Start / Stop codon** | **IN** |
| --- | --- | --- | --- | --- | --- |
| *trnQ* | N | 1-69 | 69 |  | 43 |
| *trnI* | J | 113-275 | 63 |  | -1 |
| *trnM* | J | 175-243 | 69 |  | 0 |
| *nad2* | J | 244-1,212 | 969 | ATA/TAA | -2 |
| *trnW* | J | 1,211-1,272 | 62 |  | -8 |
| *trnC* | N | 1,265-1,326 | 62 |  | 9 |
| *trnY* | N | 1,336-1,401 | 66 |  | 6 |
| *cox1* | J | 1,408-2,946 | 1,539 | ATG/TAA | -5 |
| *trnL*2^(UUR)^ | J | 2,942-3,007 | 66 |  | 0 |
| *cox2* | J | 3,008-3,677 | 670 | ATA/T-- | 0 |
| *trnK* | J | 3,678-3,749 | 72 |  | -1 |
| *trnD* | J | 3,749-3,808 | 60 |  | -3 |
| *atp8* | J | 3,806-3,958 | 153 | TTG/TAA | -7 |
| *atp6* | J | 3,952-4,587 | 636 | ATG/TAG | 2 |
| *cox3* | J | 4,590-5,369 | 780 | ATG/TAA | 5 |
| *trnG* | J | 5,375-5,434 | 60 |  | 0 |
| *nad3* | J | 5,435-5,788 | 354 | ATT/TAA | 2 |
| *trnA* | J | 5,791-5,850 | 60 |  | -2 |
| *trnR* | J | 5,849-5,913 | 65 |  | -2 |
| *trnN* | J | 5,912-5,974 | 63 |  | -1 |
| *trnS*1^(AGN)^ | J | 5,974-6,040 | 67 |  | 0 |
| *trnE* | J | 6,041-6,105 | 65 |  | 4 |
| *trnF* | N | 6,110-6,170 | 61 |  | -1 |
| *nad5* | N | 6,170-7,846 | 1,677 | TTG/TAG | 0 |
| *trnH* | N | 7,847-7,906 | 60 |  | 4 |
| *nad4* | N | 7,911-9,212 | 1,302 | ATG/TAA | -7 |
| *nad4L* | N | 9,206-9,481 | 276 | ATG/TAA | 14 |
| *trnT* | J | 9,496-9,556 | 61 |  | 0 |
| *trnP* | N | 9,557-9,616 | 60 |  | 2 |
| *nad6* | J | 9,619-10,104 | 486 | ATT/TAA | -1 |
| *Cytb* | J | 10,104-11,237 | 1,134 | ATG/TAG | -2 |
| *trnS*2^(UCN)^ | J | 11,236-11,299 | 64 |  | -10 |
| *nad1* | N | 11,290-12,231 | 942 | ATA/TAA | 0 |
| *trnL*1^(CUN)^ | N | 12,232-12,295 | 64 |  | 0 |
| *lrRNA* | N | 12,296-13,460 | 1,165 |  | 0 |
| *trnV* | N | 13,461-13,521 | 61 |  | 0 |
| *srRNA* | N | 13,522-14,228 | 707 |  | 0 |
| CR |  | 14,229-15,174 | 946 |  | 0 |

**Table S11 Mitochondrial genome organization of *Batracomorphus matsumurai***

| **Gene** | **Strand** | **Position** | **Size (bp)** | **Start / Stop codon** | **IN** |
| --- | --- | --- | --- | --- | --- |
| *trnI* | J | 1-63 | 63 |  | -3 |
| *trnQ* | N | 61-129 | 69 |  | -1 |
| *trnM* | J | 129-197 | 69 |  | 0 |
| *nad2* | J | 198-1,166 | 969 | ATT/TAA | -1 |
| *trnW* | J | 1,166-1,229 | 64 |  | -8 |
| *trnC* | N | 1,222-1,287 | 66 |  | -1 |
| *trnY* | N | 1,287-1,349 | 63 |  | 9 |
| *cox1* | J | 1,359-2,897 | 1,539 | ATG/TAA | -5 |
| *trnL2*^(UUR)^ | J | 2,893-3,961 | 69 |  | 0 |
| *cox2* | J | 2,962-3,631 | 670 | ATT/T-- | 0 |
| *trnK* | J | 3,632-3,702 | 71 |  | 1 |
| *trnD* | J | 3,704-3,764 | 61 |  | -3 |
| *atp8* | J | 3,762-3,911 | 150 | TTG/TAA | -4 |
| *atp6* | J | 3,908-4,543 | 636 | ATA/TAA | 2 |
| *cox3* | J | 4,546-5,323 | 778 | ATG/T-- | 0 |
| *trnG* | J | 5,324-5,382 | 59 |  | 0 |
| *nad3* | J | 5,383-5,736 | 354 | ATT/TAA | -1 |
| *trnA* | J | 5,736-5,797 | 62 |  | 6 |
| *trnR* | J | 5,804-5,866 | 63 |  | -1 |
| *trnN* | J | 5,866-5,929 | 64 |  | -1 |
| *trnS*1^(AGN)^ | J | 5,929-5,995 | 67 |  | 2 |
| *trnE* | J | 5,998-6,062 | 65 |  | 1 |
| *trnF* | N | 6,064-6,126 | 63 |  | 0 |
| *nad5* | N | 6,127-7,804 | 1,678 | ATT/T-- | -3 |
| *trnH* | N | 7,802-7,863 | 62 |  | -20 |
| *nad4* | N | 7,844-9,145 | 1,302 | ATA/TAA | 11 |
| *nad4L* | N | 9,157-9,429 | 273 | ATG/TAA | 2 |
| *trnT* | J | 9,432-9,495 | 64 |  | 0 |
| *trnP* | N | 9,496-9,561 | 66 |  | 2 |
| *nad6* | J | 9,564-10,049 | 486 | ATA/TAA | -1 |
| *Cytb* | J | 10,049-11,179 | 1,131 | ATG/TAG | -2 |
| *trnS2*^(UCN)^ | J | 11,178-11,240 | 63 |  | -7 |
| *nad1* | N | 11,234-12,175 | 942 | ATA/TAA | -3 |
| *trnL*1^(CUN)^ | N | 12,173-12,239 | 67 |  | 0 |
| *lrRNA* | N | 12,240-13,409 | 1,170 |  | 0 |
| *trnV* | N | 13,410-13,471 | 62 |  | 0 |
| *srRNA* | N | 13,472-14,187 | 716 |  | 0 |
| CR |  | 14,188-15,009 | 822 |  | 0 |

**Table S12 Mitochondrial genome organization of *Batracomorphus nigromarginattus***

| **Gene** | **Strand** | **Position** | **Size (bp)** | **Start / Stop codon** | **IN** |
| --- | --- | --- | --- | --- | --- |
| *trnQ* | N | 1-69 | 69 |  | 43 |
| *trnI* | J | 113-275 | 63 |  | -1 |
| *trnM* | J | 175-242 | 68 |  | 0 |
| *nad2* | J | 243-1,211 | 969 | ATA/TAA | -2 |
| *trnW* | J | 1,210-1,270 | 61 |  | -8 |
| *trnC* | N | 1,263-1,322 | 60 |  | 7 |
| *trnY* | N | 1,330-1,395 | 66 |  | 10 |
| *cox1* | J | 1,406-2,944 | 1,539 | ATG/TAA | -5 |
| *trnL*2^(UUR)^ | J | 2,940-3,005 | 66 |  | 0 |
| *cox2* | J | 3,006-3,675 | 670 | ATA/T-- | 0 |
| *trnK* | J | 3,676-3,747 | 72 |  | -1 |
| *trnD* | J | 3,747-3,807 | 61 |  | -3 |
| *atp8* | J | 3,805-3,957 | 153 | TTG/TAA | -7 |
| *atp6* | J | 3,951-4,586 | 636 | ATG/TAA | 2 |
| *cox3* | J | 4,589-5,368 | 780 | ATG/TAA | 7 |
| *trnG* | J | 5,376-5,436 | 61 |  | 0 |
| *nad3* | J | 5,437-5,790 | 354 | ATA/TAA | 7 |
| *trnA* | J | 5,798-5,857 | 60 |  | -2 |
| *trnR* | J | 5,856-5,920 | 65 |  | -2 |
| *trnN* | J | 5,919-5,981 | 63 |  | -1 |
| *trnS*1^(AGN)^ | J | 5,981-6,047 | 67 |  | 5 |
| *trnE* | J | 6,053-6,117 | 65 |  | 0 |
| *trnF* | N | 6,118-6,180 | 63 |  | 0 |
| *nad5* | N | 6,181-7,858 | 1,678 | ATT/T-- | -3 |
| *trnH* | N | 7,856-7,917 | 62 |  | 3 |
| *nad4* | N | 7,921-9,222 | 1,302 | ATG/TAA | -7 |
| *nad4L* | N | 9,216-9,491 | 276 | ATG/TAA | 10 |
| *trnT* | J | 9,502-9,562 | 61 |  | 0 |
| *trnP* | N | 9,563-9,625 | 63 |  | 2 |
| *nad6* | J | 9,628-10,113 | 486 | ATT/TAA | -1 |
| *Cytb* | J | 10,113-11,246 | 1,134 | ATG/TAA | 2 |
| *trnS*2^(UCN)^ | J | 11,249-11,311 | 63 |  | -10 |
| *nad1* | N | 11,302-12,243 | 942 | ATT/TAA | 1 |
| *trnL*1^(CUN)^ | N | 12,245-12,308 | 64 |  | 0 |
| *lrRNA* | N | 12,309-13,484 | 1,176 |  | 0 |
| *trnV* | N | 13,485-13,545 | 61 |  | 0 |
| *srRNA* | N | 13,546-14,254 | 709 |  | 0 |
| CR |  | 14,255-15,185 | 931 |  | 0 |

**Table S13 Mitochondrial genome organization of *Batracomorphus notatus***

| **Gene** | **Strand** | **Position** | **Size (bp)** | **Start / Stop codon** | **IN** |
| --- | --- | --- | --- | --- | --- |
| *trnI* | J | 1-62 | 62 |  | -3 |
| *trnQ* | N | 60-128 | 69 |  | -1 |
| *trnM* | J | 128-195 | 68 |  | 0 |
| *nad2* | J | 196-1,161 | 966 | ATT/TAA | -2 |
| *trnW* | J | 1,160-1,224 | 65 |  | -8 |
| *trnC* | N | 1,217-1,277 | 61 |  | -1 |
| *trnY* | N | 1,277-1,342 | 66 |  | -2 |
| *cox1* | J | 1,341-2,879 | 1,539 | ATG/TAA | -5 |
| *trnL2*^(UUR)^ | J | 2,875-2,941 | 67 |  | 0 |
| *cox2* | J | 2,942-3,608 | 667 | ATT/T-- | 0 |
| *trnK* | J | 3,609-3,680 | 72 |  | -1 |
| *trnD* | J | 3,680-3,741 | 62 |  | -3 |
| *atp8* | J | 3,739-3,888 | 150 | TTG/TAA | -4 |
| *atp6* | J | 3,885-4,520 | 636 | ATA/TAA | 0 |
| *cox3* | J | 4,521-5,298 | 778 | ATG/T-- | 0 |
| *trnG* | J | 5,299-5,357 | 59 |  | 0 |
| *nad3* | J | 5,358-5,711 | 354 | ATT/TAA | -1 |
| *trnA* | J | 5,711-5,771 | 61 |  | -2 |
| *trnR* | J | 5,770-5,833 | 64 |  | -2 |
| *trnN* | J | 5,832-5,894 | 63 |  | -1 |
| *trnS*1^(AGN)^ | J | 5,894-5,960 | 67 |  | 4 |
| *trnE* | J | 5,965-6,025 | 61 |  | 5 |
| *trnF* | N | 6,031-6,093 | 63 |  | -1 |
| *nad5* | N | 6,093-7,769 | 1,677 | TTG/TAG | 0 |
| *trnH* | N | 7,770-7,834 | 65 |  | 9 |
| *nad4* | N | 7,844-9,145 | 1,302 | ATG/TAA | -7 |
| *nad4L* | N | 9,139-9,414 | 276 | ATG/TAA | 2 |
| *trnT* | J | 9,417-9,478 | 62 |  | 0 |
| *trnP* | N | 9,479-9,544 | 66 |  | 2 |
| *nad6* | J | 9,547-10,029 | 483 | ATC/TAA | -1 |
| *Cytb* | J | 10,029-11,162 | 1,134 | ATG/TAA | 0 |
| *trnS2*^(UCN)^ | J | 11,163-11,224 | 62 |  | -1 |
| *nad1* | N | 11,224-12,165 | 942 | ATA/TAA | -10 |
| *trnL*1^(CUN)^ | N | 12,156-12,220 | 65 |  | 0 |
| *lrRNA* | N | 12,221-13,389 | 1,169 |  | 0 |
| *trnV* | N | 13,390-13,454 | 65 |  | 0 |
| *srRNA* | N | 13,455-14,157 | 703 |  | 0 |
| CR |  | 14,158-15,015 | 858 |  | 0 |

**Table S14 Mitochondrial genome organization of *Batracomorphus rinkihonis***

| **Gene** | **Strand** | **Position** | **Size (bp)** | **Start / Stop codon** | **IN** |
| --- | --- | --- | --- | --- | --- |
| CR |  | >1-734 | >764 |  |  |
| *trnI* | J | 765-826 | 62 |  | 0 |
| *trnQ* | N | 824-892 | 69 |  | -3 |
| *trnM* | J | 892-959 | 68 |  | -1 |
| *nad2* | J | 960-1,925 | 966 | ATT/TAA | 0 |
| *trnW* | J | 1,924-1,989 | 66 |  | -2 |
| *trnC* | N | 1,982-2,043 | 62 |  | -8 |
| *trnY* | N | 2,043-3,106 | 64 |  | -1 |
| *cox1* | J | 2,105-3,643 | 1,539 | ATG/TAA | -2 |
| *trnL2*^(UUR)^ | J | 3,639-3,704 | 66 |  | -5 |
| *cox2* | J | 3,705-4,371 | 667 | ATT/T-- | 0 |
| *trnK* | J | 4,372-4,443 | 72 |  | 0 |
| *trnD* | J | 4,444-4,507 | 64 |  | 0 |
| *atp8* | J | 4,505-4,654 | 150 | TTG/TAA | -3 |
| *atp6* | J | 4,651-5,286 | 636 | ATA/TAA | -4 |
| *cox3* | J | 5,287-6,064 | 778 | ATG/T-- | 0 |
| *trnG* | J | 6,065-6,123 | 59 |  | 0 |
| *nad3* | J | 6,124-6,477 | 354 | ATC/TAA | 0 |
| *trnA* | J | 6,477-6,537 | 61 |  | -1 |
| *trnR* | J | 6,536-6,599 | 64 |  | -2 |
| *trnN* | J | 6,598-6,659 | 62 |  | -2 |
| *trnS*1^(AGN)^ | J | 6,659-6,725 | 67 |  | -1 |
| *trnE* | J | 6,741-6,804 | 64 |  | 15 |
| *trnF* | N | 6,809-6,875 | 67 |  | 4 |
| *nad5* | N | 6,875-8,554 | 1,680 | ATT/TAG | -1 |
| *trnH* | N | 8,552-8,614 | 63 |  | -3 |
| *nad4* | N | 8,619-9,920 | 1,302 | ATG/TAA | 4 |
| *nad4L* | N | 9,914-10,189 | 276 | ATG/TAA | -7 |
| *trnT* | J | 10,192-10,253 | 62 |  | 2 |
| *trnP* | N | 10,254-10,318 | 65 |  | 0 |
| *nad6* | J | 10,321-10,803 | 483 | ATT/TAA | 2 |
| *Cytb* | J | 10,803-11,936 | 1134 | ATG/TAA | -1 |
| *trnS2*^(UCN)^ | J | 11,936-11,998 | 63 |  | -1 |
| *nad1* | N | 11,998-12,933 | 936 | ATA/TAA | -1 |
| *trnL*1^(CUN)^ | N | 12,931-12,995 | 65 |  | -3 |
| *lrRNA* | N | 12,996-14,167 | 1,172 |  | 0 |
| *trnV* | N | 14,168-14,232 | 65 |  | 0 |
| *srRNA* | N | 14,233-14,970 | 738 |  | 0 |
| CR |  | 14,971->15,385 | >415 |  | 0 |
