# Supplementary material for: The mitochondrial genome sequences of eleven leafhopper species of Batracomorphus (Hemiptera: Cicadellidae: Iassinae) reveal new gene rearrangements and phylogenetic implications
Source: PeerJ. 2024 Oct 22;12:e18352. doi: 10.7717/peerj.18352 (PMC11505954; doi:10.7717/peerj.18352)
Supplement: Table S15 — Note: B. rinkihonis indicates Batracomorphus rinkihonis, B. notatus indicates Batracomorphus. B. notatus , B. nigromarginattus indicates Batracomorphus nigromarginattus, B. matsumurai indicates Batracomorphus matsumurai, B. lineatus indicates Batracomorphus lineatus, B. fuscomaculatus indicates Batracomorphus fuscomaculatus, B. extentus indicates Batracomorphus extentus, B. curvatus indicates Batracomorphus curvatus, B. cornutus indicates Batracomorphus cornutus, B. chlorophana indicates Batracomorphus chlorophana, B. allionii indicates Batracomorphus allionii. [file peerj-12-18352-s012.docx]

**Table S14.** Collection information of specimen in the present study.

| **Name** | **Locality** | **Collecter** | **Time** |
| --- | --- | --- | --- |
| *B. rinkihonis* | Taohuachong, Yingshan, Hubei (N. 30°12’, E. 115°5’6’’) | Qu-Ling | 23, Jun 2014 |
| *B. notatus* | Baihua Mountain, Baoshan, Yunnan (N. 25°12’, E. 99°18’) | Wang-Jiajia, Zhang-Chao | 23, May 2019 |
| *B. nigromarginattus* | Qingtaiguan, Luotian, Hubei (N. 31°09’, E. 115°43’) | Qu-Ling | 03, Jul 2014 |
| *B. matsumurai* | Shenlongjia, Hubei (N. 31°25’20’’, E. 110°44’) | Zhong-Likun | 14, Jul 2018 |
| *B. lineatus* | Tongbiguan, Yingjiang, Yunnan (N. 26°10’, E. 98°38’) | Wang-Xianyi, Zuo-Qin | 31, May 2019 |
| *B. fuscomaculatus* | Huanglian Mountain, Luchun, Yunnan (N. 22°28’, E. 102°24’) | Wang-Jiajia, Zhang-Chao | 09, Jun 2019 |
| *B. extentus* | Huaping, Guangxi (N. 25°34’, E. 109°48’) | Wang-Xianyi | 05, May 2017 |
| *B. curvatus* | Huaping, Guangxi (N. 25°34’, E. 109°48’) | Wang-Xianyi | 05, May 2017 |
| *B. cornutus* | Longgang, Longzhou, Guangxi (N. 22°25’, E. 106°45’) | Qu-Ling | 05, May 2014 |
| *B. chlorophana* | Wuzhi Mountain, Hainan (N. 18°38’, E. 109°26’) | Yan-Bin | 17, Apr 2017 |
| *B. allionii* | Tianma National Nature Reserve, Luan, Anhui (N. 31°15’, E. 115°21’) | Zhong-Likun | 23, Jul 2018 |

Note: *B. rinkihonis indicates Batracomorphus rinkihonis, B. notatus indicates Batracomorphus. B. notatus, B. nigromarginattus indicates Batracomorphus nigromarginattus, B. matsumurai indicates Batracomorphus matsumurai, B. lineatus indicates Batracomorphus lineatus, B. fuscomaculatus indicates Batracomorphus fuscomaculatus, B. extentus indicates Batracomorphus extentus, B. curvatus indicates Batracomorphus curvatus, B. cornutus indicates Batracomorphus cornutus, B. chlorophana indicates Batracomorphus chlorophana, B. allionii indicates Batracomorphus allionii.*
